# Porous Crumb Structure of Leavened Baked Products

**DOI:** 10.1155/2018/8187318

**Published:** 2018-08-05

**Authors:** H. A. Rathnayake, S. B. Navaratne, C. M. Navaratne

**Affiliations:** ^1^Department of Food Science and Technology, Faculty of Applied Sciences, University of Sri Jayewardenepura, Gangodawila, Nugegoda, Sri Lanka; ^2^Department of Agricultural Engineering, Faculty of Agriculture, University of Ruhuna, Mapalana, Kamburupitiya, Sri Lanka

## Abstract

Quality evaluation of the porous crumb structure of leavened baked goods, especially bread, has become a vast study area of which various research studies have been carried out up to date. Here is a brief review focusing on those studies with six main parts including porous crumb structure development, crumb cellular structure analysis, application of fractal dimension for evaluating crumb cellular structure, mechanical and sensorial properties of crumb structure, changes of porous crumb structure with staling, and modifications to obtain a well-developed porous crumb structure and retard staling. Development of the porous crumb structure mainly depends on dough ingredients and processing conditions. Hence, certain modifications for those factors (incorporating food hydrocolloids, emulsifiers, improvers, etc.) have been conducted by cereal sciences for obtaining well-developed porous crumb structure and retard staling. Several image analysis methods are available for analyzing microstructural features of porous crumb structure, which can directly affect the mechanical and sensorial properties of the final product. A product with a well-developed porous crumb structure may contain the property of higher gas retention capacity which results in a product with increased volume and reduced crumb hardness with appealing sensorial properties.

## 1. Introduction

Leavened baked products include a wide range of food products, such as bread, buns, and cakes that are commonly consumed throughout the world over the past 150 years [1]. Even though foods have similar chemical composition, they may exhibit different mechanical behavior and sensorial properties depending on their cellular structure [2, 3]. Hence, the quality parameters of leavened baked products are mainly related to crumb's mechanical and sensorial properties that may influence consumer purchase [3, 4].

Crumb grain has been defined in literature as the exposed cell structure of crumb when a leavened baked product is sliced [5–7] which can be generally seen as a two-phase soft cellular solid, consisting of a solid phase apparent in the cell wall structure and a fluid phase made up of air [8, 9]. According to materials science approach, solid cellular materials can be categorized basically as open or closed cell foam. Open cell structured porous food materials consist of pores that are connected to each other through an interconnected network [10, 11], which is comparatively softer than closed cell foam structures [11]. The cell foam that does not have interconnected pores is considered as closed cell forms [10, 11] and contains higher compressive strength due to the dense structures [11].

Due to the complex mechanical behavior of crumb structure [8, 12–15], close examination of different slices can reveal considerable variation in the cell characteristics even within a single sample [13]. Hence, vast range of research studies have been carried out through decades for understanding the structure and properties of the crumb structure with regard to the mechanical and sensorial quality of the final product [2, 5, 12, 16–18].

The purpose of this review is to identify and summarize the literature that covers the characteristics and development of the porous structure of dough and how it can affect the physical and sensorial quality of the final product. The most commonly concerned properties include product volume [19, 20], texture [4, 6, 8], and cellular structural properties. Further, this review covers some literature of physicochemical changes of porous crumb structure during storage period (crumb staling) and certain research studies carried out with a view to improve the porous crumb structure and retard the staling process.

## 2. Porous Crumb Structure Development

Development of porous crumb structure mainly depends on dough ingredients, processing conditions [5, 8, 11, 12, 19, 21], yeast activity, fermentation temperature, and gas bubble formation [21, 22].

The basic ingredients that are used for a leavened baked product are flour, water, leavening agent (either a chemical leavening agent like NaHCO_3_ or biological leavening agent like yeast), NaCl [8, 10], sugar, and shortening. There are number of processes to convert the ingredients into a well-developed porous structure, where the main processing steps involve kneading, fermentation, proofing, and baking.

Water and flour are the most significant ingredients which may affect considerably the texture and crumb properties [1].

Wheat flour is the most commonly used flour type for leavened baked products made up of a mixture of two groups of proteins named gliadins and glutenins [23, 24]. During mixing and hydration, these two proteins combine together and form a viscoelastic gluten network that can retain leavened gas during fermentation and baking [23, 25–27]. The starch associated with this gluten network (rather the moistened starch) becomes gelatinized during heating and form a semirigid structure to the product along with the coagulated gluten (gluten protein gets denatured during heating and protein-protein crosslinking occurs via formation of disulfide bond) [11, 28, 29]. Additionally, Rouillé et al. [30] stated that the soluble fraction of wheat flour affects both loaf volume and crumb fineness in an opposite way. According to the research conducted by He and Hoseney [31], selection of flour with better protein quality may result in a product with better porous structure with uniform sized gas cells. Poorly built gluten network may fail to retain leavened gas that results in a product with lower loaf volume.

Approximately 50% water results in a finely textured, light crumb, and a dough prepared with a higher water percentages may result in a coarser crumb with more carbon dioxide (CO_2_) [1].

Two forms of yeast are being used for leavened baked products naming, moist pressed cakes and dehydrated granules both of which consist of billions of living cells of* Saccharomyces cerevisiae* [14, 28]. When wheat flour was rehydrated, the yeast begins to metabolize and ferment producing CO_2_ as a by-product (the mechanism of yeast fermentation will be described in a latter part of this review). When using a wheat flour type with a little gluten development ability (E.g., cake flour), baking powder (chemical leavening agents) may be used. If a biological leavening agent is used, gas evolving rate becomes high and leavened gas will largely escape from the batter. Hence the gas cells may overexpand and may lead to collapse resulting in a coarse-grain structure with lowered volume [28].

Brooker [32] had mentioned that the addition of small amount of shortening to the dough may lead to improve loaf volume and results in finer and more uniform crumb structure within cell walls. Further, Brooker [32] has found that the addition of crystal fat is far better than addition of oil. When adding shortening to the dough mix, fat crystals are ejected from shortenings during mixing, become enveloped by a fat (crystal)–water interface, and are able to stabilise large numbers of small air bubbles by adsorbing to their surface. During baking, air bubbles can expand without rupturing because of extra interfacial material provided by adsorbed fat crystals when they melt which result in a product with fine crumb structure [28, 32].

Sugar may act as a tenderizer, sweetener, and additional fermentable substrate. And also sugar has moisture retaining properties of baked goods [28]. Additionally, sugar has the ability to increase starch gelatinization and protein denaturation temperatures, which may lead to improve air bubble expansion during baking [11, 33].

Kneading process may lead the ingredients to get mixed homogeneously, to absorb water by hydrophilic groups of flour protein molecules, for the development of gluten protein, and to build up a viscoelastic structure and entrainment of air into the dough mass [8, 12, 14, 23, 25–28]. Certain researches have mentioned that the nuclei for gas cell development can be generated during the mixing process within the air phase of the dough [8, 12, 14, 23].

During fermentation, the yeast cells utilize the carbohydrates in the absence of oxygen (since dough making is reported as an anaerobic process) to produce energy, alcohol (ethanol), and CO_2_ as the end-products [12, 34, 35] via a series of intermediate stages, in which many enzymes take part. Apart from that, fermentation process is also important for formation of flavor substances [14, 35].

The generated CO_2_ may partly dissolve within the liquid phase and diffuses to the nuclei generated during mixing stage due to concentration gradient of gas [8, 12, 31, 36–38] that causes the modifications of the dough structure causing physicochemical changes in gluten network and other proteins giving the characteristic porosity of the porous crumb [22, 37]. When CO_2_ defused to the nuclei in the liquid phase, the nuclei may expand into gas cells [8, 31, 39] and the density of the dough can be reduced [8] while increasing the pressure a little [31]. He and Hoseney [31] had stated that the pressure inside the gas cells could be slightly greater than that of the atmospheric pressure which had been reported as 1.01 atm. This small pressure increment (0.01atm) had occurred due to the result of surface tension at the gas-dough interface and the viscous resistance of the dough to expansion.

The process of carbohydrate fermentation is scientifically known as the tricarboxylic acid cycle (TCA) [35]. Baker's yeast can ferment all the main types of sugars in dough including glucose, fructose, saccharose (sucrose), and maltose [14, 35]. Glucose and fructose become fermented immediately. After almost all the built-up fructose and glucose have been depleted, sucrose is first converted to glucose and fructose by the enzyme amylase [28, 35, 40]. This latter process occurs very rapidly, and a few minutes after mixing the dough, all sucrose molecules have been converted into glucose and fructose. Maltose molecules may be hydrolyzed to two molecules of glucose with the help of the yeast enzyme maltase [14, 35, 41]. The simplified equation of dough fermentation can be indicated as mentioned in the following equation.

(1) $$\begin{array}{c} C_{6}H_{12}O_{6}⟶2C_{2}H_{2}OH+2{CO}_{2}+234kJ \end{array}$$

The amount of carbon dioxide produced in dough by sugar fermentation may amount to about 70% of the theoretical amount indicated by the chemical equation. This can be explained by the fact that part of the sugar is used for energy and reproduction of the yeast cells within the dough [35]. Dough expansibility during fermentation can be mainly determined by viscoelasticity. The viscous components in the dough mass allow gas cells to expand to equalize the pressure, whereas the dough elastic components provide the relevant strength to prevent the dough from overexpansion and collapsing [31].

Heat and mass transfer phenomena are taking place simultaneously during bread baking which causes physical, chemical, and structural transformation [1] including water evaporation, volume expansion, starch gelatinization, and protein denaturation, settling the porous structure [1] which leads to setting the final bread crumb structure within the oven [8]. Usually, the protein denaturation and starch gelatinization occur during the temperature interval of 60–85°C and contribute to the change from dough to crumb [1]. With the temperature increment within the oven, thermal expansion of vapor occurs and the saturation pressure of water within the dough get increased. This causes the loaf to be expanded (oven spring). According to Hayman, Hoseney, and Faubion [42], the loaf expansion occurs by increasing the product volume during the first 6-8 min in baking, creating a high strain within the dough that can compress the heat set cellular structure of the outer reagents of the product [1, 8, 31, 43]. As a result of that, the outer cells can be elongated with their long axes parallel to the crust planes [8]. CO_2_ also plays an important role in the expansion of bubbles during baking by releasing from the dough when the bubble walls start to break under pressure, making the porous structure more continuous and open to the outside of the bread [1].

When analyzing the crumb structure, several factors can be taken into account and the most common factors considered in most researches are crumb appearance, product volume [8, 44–50], resilience of the product [8], crumb color [8, 9, 16, 38], consumer appeal [2, 8, 11, 16, 51–53], physical texture of the product [7–9, 46, 54–57], taste [8, 9], compactness and uniformity of the crumb grains [58], size, shape, uniformity, and wall thickness of crumb cells or pores [2, 7, 8, 16, 17, 30, 58, 59].

## 3. Evaluating Crumb Cellular Structure

Recently, image analysis (IA) has been used as a quantitative tool that provides directly interpretable data for reliably assessing the microstructural features of crumb and its relationship with the crumb mechanical and sensorial properties [1, 2, 7, 10, 41, 44] of the final product as well as the crumb structure evaluation during fermentation and baking [22]. The most common features that can be analyzed using digital image analysis can be considered mainly as cell size, cell size distribution, number of cells per unit area, cell wall thickness, void fraction (porosity), shape factor, and number of missing cell walls [2, 8, 13, 16, 17, 30, 41, 59].

Image analysis involves several steps including image acquisition, image preprocessing, image segmentation, feature extraction, and classification [38]. Three elements are reported to be required to acquire the digital image of cut surface of a porous crumb including a source of illumination, the specimen, and an image sensing device [60].

There are certain methods that have been used for image acquisition, among which light microscopy and electron microscopy [2, 61] had been recorded as the most convenient imaging techniques applied to food structure analysis [2]. Apart from that, digital scanners and conventional photography [5, 21, 22, 38, 58] had been commonly used to capture two-dimensional (2D) high-resolution images of porous crumb structure and have been recommended as fast, convenient, economically feasible, and robust methods that provide good accuracy by acting independently from external light [3, 5, 38, 58]. There are some more advanced high-resolution techniques also available for the purpose of providing quantitative information of the porous crumb structure [21, 22], such as scanning electron microscopy [5, 14, 21, 22], X-ray computed tomography [2, 3, 10, 11, 14, 21, 22, 38, 62, 63], and magnetic resonance imaging [3, 5, 11, 14, 21, 22, 38, 64, 65].

**Figure 1:**
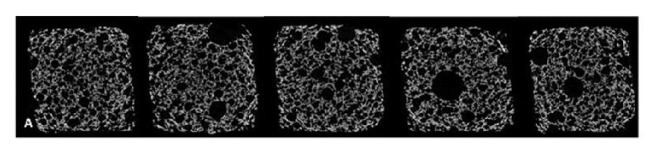
X-ray microtomography 2D reconstructed cross section images of cake samples [11].

X-ray computerized microtomography (X-MCT) had been the high spatial resolution version of the computed axial tomography routinely used for medical diagnosis and been applied for porous crumb structure analysis in several studies [2, 10, 11, 62, 63, 66].  Figure 1 represents an example of X-ray microtomography 2D reconstructed cross section images of cake samples from the research done by Sozer et al. [11]. X-MCT provides the ability to obtain three-dimensional (3D) representation of the inside structure of a sample from a set of projection measurements recorded from a certain number of points of view and examine their textural characteristics [2, 10, 11]. Mathieu et al. [62] had mentioned that the kinetics of bubble growth and foam setting in dough during fermentation and proofing can be determined by this method. The main disadvantage of X-MCT is the poor intrinsic contrast of low-density materials or porous crumb structures [2].

**Figure 2:**
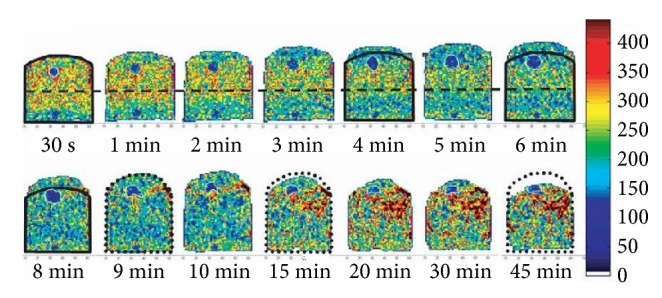
Normalized magnetic resonance images (MRI) of bread acquired during baking. That is, during baking, the cold colors convert to warm colors corresponding to low to high signal intensities, respectively [65].

Several research studies have been carried out up to date regarding porous crumb structure evaluation by using magnetic resonance imaging (MRI) [21, 64, 65, 71]. Among them, Wagner et al. [65] had used a spacious MRI oven compatible with a low-field MRI scanner (0.2 T) to monitor bread loaf fermentation and baking.  Figure 2 shows normalized magnetic resonance images of bread taken within that oven during baking. Bajd and Serša [21] have proved that the use of high-field MRI scanners with magnetic resonance microscopy (MRM) can overcome the resolution problem obtained in low-field MRI experiments (because even though those low-field methods have good temporal resolution and image quality, they are lacking in spatial resolution). The advantages of applying MRI in image analysis have been recorded in literature as noninvasiveness, ability to determine precise moisture content, and containing a comparatively high spatial resolution [14, 21, 65].

### 3.1. Image Segmentation

Image segmentation can be considered as a method that separates object(s) of interest within an image from its background, typically yielding a binary image [5, 8, 16]. This segmentation process is important in crumb structure analysis to accurately segregate the gas and solid phases and define the distribution of cell and cell wall sizes [7, 8]. The most common way of segmenting images had been recognized as thresholding and edge detection [5, 7, 8, 30].

**Figure 3:**
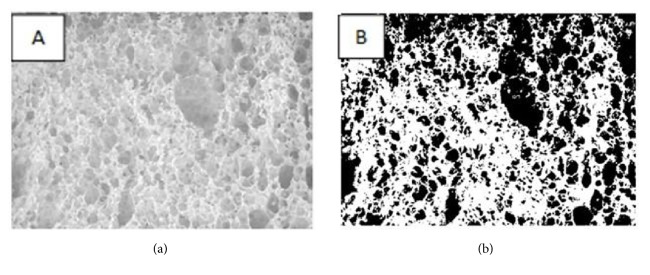
Original (2D) Gray Image (a) and segmented (b) gas cells where black pixels represent bubbles and white pixels represent the porous structure [18].

Thresholding can be either subjective (chosen manually) or objective. The objective method is based on statistical techniques referred to as clustering and it is considered as one of the most commonly used techniques for optimal thresholding [5, 8, 30]. Literature stated that the using of single threshold value for image segmentation of porous crumb structure may lead to under- and overestimation of cell sizes due to nonuniform structure of the bread crumb [8]. Hence more sophisticated thresholding techniques such as multiple thresholding had been recommended. Scanlon and Zghal [8] had described an example for this phenomenon as a local segmentation where there is application of neighborhood of pixels to detect individual objects within an image or individual crumb cells using gray level threshold for each crumb cell.  Figure 3 shows an example for digital image segmentation. The scanned images of porous crumb structure can be threshold and analyzed by different scientific image analysis software which has been developed by numerous researchers utilizing multiple algorithms from cell segmentation techniques that aim at determining the cell size and shape distribution [5, 13, 67]. An example list of software is mentioned in Table 1.

**Figure 4:**
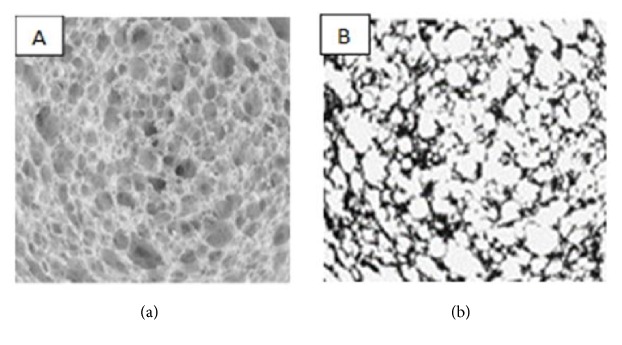
Original 2D Gray Image (a) and segmented (b) gas cells using K-means algorithm [44].

Before introducing scientific image analysis software, image segmentation has been done following cluster analysis method which was commonly known as the* K*-means algorithm which was mostly used for classifying digitized images into cells and background [3, 7]. In general, the algorithm groups a set of data that contains* M* observations described by* N* variables or features into* K* clusters [7]. This algorithm adapts the gray level thresholding of each bread slice image, depending on the overall brightness of the crumb image and the distribution of constituent pixel gray levels, both of which can be affected by the crumb structure itself [44]. The corresponding segmented images appear to provide an accurate binary representation of the complex cellular structure seen in the original gray-scale images.  Figure 4 represents a comparison of an original and segmented image with K-means algorithm.

Crumb fineness or cell density is the value determined by the total number of cells detected over total surveyed area. And the ratio between the numbers of cells lower than 1 mm to the number of cells higher than 1 mm in diameter gives the cell uniformity which is reported to be directly correlated to fineness [3, 5, 30, 67, 73]. Cell density can strongly influence the mechanical properties of bread crumb [67]. Che Pa et al. [5] mentioned that higher value of crumb fineness indicates a finer crumb structure.

Cell wall thickness (*μ*m) is determined on the cubic subvolumes (100 × 100 × 100 pixels) randomly extracted from the considered whole volume [2]. According to Scanlon and Zghal [8], thickness of the cell wall depends on the differences in starch content (e.g., thinner cell wall may result due to availability of lesser amount of starch granules) and moisture content of the dough mass. Further, Scanlon and Zghal [8] described that thinner cell walls may cause greater mechanical strength and greater deflection at break (i.e., the flexibility of thinner cell walls is higher compared to thicker cell walls) and also cause crumbs to be softer [47].

When the crumb structure rises during proofing, defects in the cell walls (missing due to coalescence or ruptured cell walls) and variability in cell wall distribution are some factors that must be considered in microstructure analysis of the porous crumb structure [8]. This has been considered in literature as the missing cell walls. Zghal, Scanlon, and Sapirstein [20] had derived an equation (2) to calculate the number of missing cell walls by calculating the theoretical number of cells/cm^2^ and the number of cells/cm^2^ determined by image analysis.

(2) $$\begin{array}{c} N_{x}=N_{0}\left(\;1-\left[\;\frac{{\left(1/\rho_{x}\right)}^{2/3}-{\left(1/\rho_{0}\right)}^{2/3}}{{\left(1/\rho_{0}\right)}^{2/3}}\;\right]\;\right) \end{array}$$

N_x_ represents the theoretical number of cells at the time t_x_. Zghal, Scanlon, and Sapirstein [20] have considered t_x_ as 35 minutes from the beginning of proofing time. N_o_ means the number of cells/cm^2^ at the highest density of the sample at t_o_ time measured by digital image analysis (DIA) and *ρ*_x_ and *ρ*_o_ are densities of the product at the time t_x_ and t_o_, respectively. Then the number of missing cell walls can then be calculated from the following equation:

(3) $$\begin{array}{c} N_{mcw}=N_{x}-N_{f} \end{array}$$

N_mcw_ represents the number of missing cell walls, and N_f_ represents the number of cells/cm^2^ of the final product determined by DIA.

Crumb porosity (void fraction) had been expressed as the mean value of the total cell to total area ratio on each slice of the considered volume. A higher void fraction suggests an increase of the number of larger cells (>1 mm diameter) and consequently a decrease in the degree of cell uniformity [5]. According to Zghal, Scanlon, and Sapirstein [44], the values of the mean cell area and void fraction have to be multiplied by a corrective factor of 1.5, because they found that, on average, the cell volume based on observation of cell size of the surface of a slice is 41% lower than the actual volume, assuming that the cells are spherical in shape and sectioned randomly to give cells with a uniform size distribution [5].

Relative density of bread crumb is a dominant physical characteristic which can affect the elastic properties and mechanical strength representing the 3D structure of cellular solids [11, 20] and is defined as the fraction of voxels segmented as cell walls. It is comparable to the ratio *ρ∗*/*ρ*_s_, where *ρ∗* represents the density of the crumb and *ρ*_s_ is the density of the material of the cell walls [3]. Zghal, Scanlon, and Sapirstein [44] have proved that, with the increment of the proofing time, gas cell coalescence may occur which will lead to nonuniformity in relative density due to missing cell walls which can weaken the crumb strength.

According to that, a product with well-developed porous crumb structure should have a high porosity and fine, regular gas cell structure [9, 74].

Apart from the undoubted advantages, Falcone et al. [2] have stated that there are some problems that occurred when imaging techniques are used on such food materials. One of them is that most of the imaging techniques require a sample preparation that can produce artifacts (e.g., especially when image analyzing using light microscopy and electron microscopy) which has to be considered to avoid wrong conclusions in microstructure investigation. Further, some imaging techniques are more expensive because they require sophisticated equipment; those can only be applied on foods having a high commercial value. Farrera-Rebollo [58] had also reviled certain problems arising in image analysis. For example, there are certain differences in the results of different image analysis methods (such as scanning resolution) even for similar products. Che Pa et al. [5] have mentioned that it is difficult to accurately determine the structure of the porous crumb structure due to the lack of uniformity in cell distribution and the higher variation in gas cell size. Mathieu et al. [62] and Lassoued et al. [3] have also mentioned the complex nature and of porous structure and difficulty in cell segmentation of 2D images and exact identification of the relationship between microstructure and mechanical properties. Hence, researches had focused more on overcoming those disadvantages in microstructure analysis of foods with complex cellular structure.

## 4. Application of Fractal Dimension for Evaluating Crumb Cellular Structure

Visual textures are generally formed by the interaction of light with a rough surface. In a digital image of a surface, information is stored as an array of pixels with different intensities or gray levels. Therefore, the local variation of brightness from one pixel to the next (or within a small region) is often called texture [75].

Image texture analysis also called texture feature is a region where there is a descriptive approach that provides a measure of properties such as smoothness, coarseness, and regularity [3, 8, 75]. Fractal dimension (FD) provides a numerical descriptor of the morphology of objects with complex and irregular structures and it is reported to be applied to explain changes in the structure of food materials during or as a consequence of processing [17, 58]. FD can be evaluated using the Box Counting Method (BCM) [13, 17, 75], Differential Fractal Brownian Motion Method (FBMM) [13, 75], the Frequency Domain Method (FDM) [13, 75], morphological fractal (M) [13], mass fractal method (MF), and spectral dimension or random walks method (RW) [13]. All these studies show that image texture analysis has potential for determining some cellular structural features, while avoiding thresholding and cell segmentation of 2D images [3].

Fractal Brownian Motion Method (FBMM) is based on the average absolute difference of pixel intensities and is an example of a statistical fractal that can be described by the Hurst coefficient [13]. In Frequency Domain Method, the fast Fourier transforms (FFT) are taken in the horizontal and vertical directions and then the FD is taken from the average value of the vertical and horizontal fractal dimensions FFT_d_. The mass fractal dimension, MF_d_, is mostly used to describe the heterogeneity and space-filling ability of an object which can be estimated from the negative slope of the logarithmic plot of number of pores, in m number of pixels versus log m [13].

Pérez-Nieto et al. [17] have clearly described the method of calculating the mean fractal dimension of the perimeter of the pores ($\overset{-}{FD}$sc) using the results obtained from ImageJ analysis and stated that resulting in a higher mean fractal dimension may indicate a more convoluted or jagged pore structure resulting in a product with a rough fractal texture. Equation (4) represents how to calculate the FDsc value and afterwards, the mean fractal dimension ($\overset{-}{FD}$sc) can be calculated by (5).

(4) FDsc=2log⁡P/4log⁡A

(5) FD−sc=∑i=1nFDscin

n represents the number of cells (objects), FDsc represents fractal dimension of the perimeter of a single cell, and P and A represent the individual cell perimeter in pixel and individual cell area in pixel, respectively.

Additionally Pérez-Nieto et al. [17] have calculated the fractural dimension of the crumb structure using the Shifting Differential Box Counting Method (FD_SDBC_) using ImageJ software which corresponds to the 2D gray level crumb images by the slope of the least-squares linear regression of the log (box count) versus log (box size) plot and by (6), where “N” is the number of boxes and “r” is the length of the side of box. Higher values of FD_SDBC_ represent more complex or rougher gray level crumb images, while low values of FD_SDBC_ can be associated with simpler or smoother images. The same test was described by Quevedo et al. [75] using Matlab 5.0 software using different food materials. Gonzales-Barron and Butler [13] have described relative differential Box Counting Method (RDBC) for calculating FD.

(6) $$\begin{array}{c} {FD}_{SDBC}=\frac{\log\left(N\right)}{\log\left(1/r\right)} \end{array}$$

## 5. Mechanical and Sensorial Properties of the Porous Crumb Structure

Crumb mechanical properties can vary microscopically and macroscopically, where the microscopic variations may occur due to the volume fraction of the granules that can be determined by the finite thickness of the cell wall. And the macroscopic variations may occur due to the variation of crumb moisture content across the product that may represent the differences in the degree of melting of starch granules [8].

Yeast fermented dough is considered to have complex mechanical behaviors, and the dimensions and physical properties of the dough may change with the time [8, 12–15]. Lack of homogeneity in the crumb cell distribution and development of complex stress combination during crumb mechanical testing are also named as the reasons for the complex mechanical behavior of bread crumbs [8]. Furthermore, invasive, continuous measurements on dough are generally not adequate as they may provoke dough collapse [12]. Hence, the choice of the most appropriate analytical procedure is thus crucial for the full comprehension of the underlying mechanisms of leavening and crumb structure development.

### 5.1. Texture Analysis

Apart from the crumb grain appearance, physical texture is also an important quality to determine the porous structure of baked products where the texture is related to the geometric and mechanical properties of the product, which heavily depends on its cellular structure [5, 8, 9, 13, 44, 55, 76] such as cell wall thickness, cell size, and uniformity [5, 8, 44] which has been defined as the cellular structure of a crumb of a slice of a product [8].

Texture profile analysis had been created as an imitative test that resembles what goes on in the human mouth and is a parameter to determine the human perception of the product's texture and how it behaved when handled and eaten. Furthermore, it incorporates all the attributes (mechanical, geometric, and surface) of the food, suggesting that the experience of texture is one of many stimuli working together in combination [77]. The most commonly considered attributes of leavened baked products include hardness, springiness, adhesiveness, chewiness, gumminess, and cohesiveness [46, 54].

**Figure 5:**
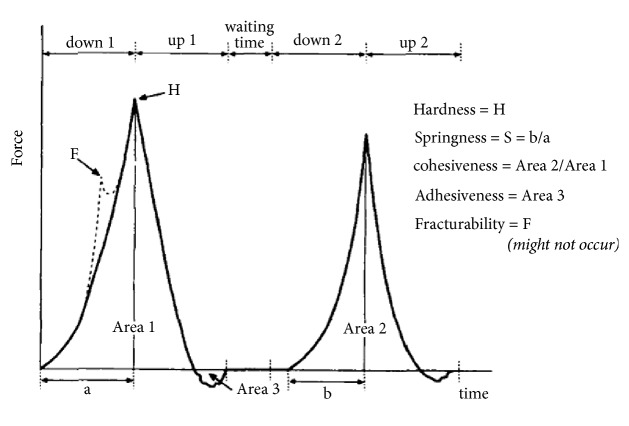
Instrumental texture profile analysis obtained with a TA-XT2 Texturometer [72].

Hardness (g) is measured from the peak force on first compression and is defined as the force required for biting bread samples. Springiness (mm) is calculated from the distance of the sample recovered after the first compression. Adhesiveness (mJ) represents the work necessary to overcome the attractive forces between the surface of food and that of the sensor during the first and second compression cycle. Cohesiveness is a characteristic of mastication that can be calculated by the ratio of the active work done under the second cycle area to the first cycle area. More cohesive dough may result in a product with higher specific volume and softer texture. Gumminess (g) depends on hardness and cohesiveness which presents the density that persists throughout chewing. Gumminess of the crumb also depends on the tenacity and extensibility of the dough and the flour protein content. Chewiness (mJ) depends on gumminess and springiness which describes how long it takes to chew a food sample to the consistency suitable for swallowing [7, 46, 54–57].  Figure 5 represents the compression curve of force versus time and the summary of obtaining the major texture parameters.

### 5.2. Sensory Evaluation

Crumb mechanical properties as well as consumer acceptability can also be determined by the application of sensory evaluation [2], because how the crumb feels by touch or in the mouth is greatly influenced by the size or the structure of the crumb cells. As an example, crumb with finer, thin-walled, uniformly sized cells yields a softer and more elastic texture than crumb with coarse and thick-walled cell structure [11, 16].

Crumb appearance, aroma, texture, taste, and degree of satisfaction can be named as the main parameters tested in sensory evaluation [48, 52]. Appearance induces the palatability and the consumer acceptability of the product. Aroma and taste of the product represents the presence of many volatile and nonvolatile components, whereas the nonvolatile compounds contribute mainly to the taste and the volatiles influence both taste and aroma [52]. According to Schieberle [53], the amount of flavor compounds formed in bread can be affected by yeast amount and activity, fermentation and baking time, and temperature [51, 52].

When evaluating the texture of the bread crumb in sensory evaluation, improved elasticity, softness, and sponginess are the main parameters considered to determine the quality of the product.

### 5.3. Dough pH

The degree of acidity is considered as a parameter that determines the physical state of the gluten, influences the growth and the activity of the yeast, and controls the growth of many other microorganisms. According to Miller, Graf, and Hoseney [78], carbon dioxide dissolves in the aqueous dough phase until it becomes saturated (especially in the early stages of the fermentation) and after that, gaseous carbon dioxide diffuses into bubbles or into the atmosphere. The dissolved carbon dioxide reacts with water to form carbonic acid which imparts the acidic pH of the dough. With the fermentation process progressing, the pH of the dough get reduced [38, 49] and should be within the range of 5.2-6.0 [79].

### 5.4. Product Volume

Product volume (cm^3^) is considered as an important bread characteristic since it provides a quantitative measurement of baking performance [1, 55] and leavened gas retention capability within the dough mass [37, 43, 69]. The desirable loaf volume of yeast fermented products is achieved only if the dough provides a favorable environment for yeast growth and gas generation. At the same time, it also represents a strong gluten matrix that is capable of maximum gas retention [12]. Zghal, Scanlon, and Sapirstein [44] and Tlapale-Valdivia et al. [38] had stated that the product volume can be affected by the dough mixing and proofing time.

Rapeseed displacement method is the most commonly used method for the determination of product volume [44–50]. Apart from that, there are some other methods also available for determination of gas production of a fermented dough such as the oven rise recorder, alveograph method, and pressure meter methods [12, 14].

### 5.5. Other Common Physical Properties

Apart from the aforementioned mechanical and sensorial properties that are most commonly carried out to determine the properties of porous crumb structure, some other parameters were also described in literature for evaluating the mechanical properties of porous crumb structure. Among them, specific volume (gcm^−3^) is an important visual characteristic for leavened baked products which could strongly influence the consumer's choice when evaluating product quality [80] and is also reported to have an effect on certain mechanical properties of the crumb structure such as crumb hardness and relative elasticity [8]. Bulk density (gcm^−3^) is mostly used to describe the density of the cellular solid [8]. The bulk density can be affected by the particle size and the density of flour or flour blends [81] and also by crumb porosity [51].

Determination of crumb permeability utilizing Darcy's law is considered as a simple tool for assessing crumb texture along with specific volume [10]. Compression testing using stress-strain pots and calculating Young's moduli by the slope of the stress-strain curve is another method mentioned in literature for evaluating porous crumb structure. Certain researches mention Young's module for characterizing crumb properties [8, 10, 20, 62] where density is the most highly correlated parameter [20]. Further, Zghal, Scanlon, and Sapirstein [20] stated that Young's modulus can be positively correlated with density, crumb brightness, cells/cm^2^, and uniformity of cell wall thickness and negatively correlated with void fraction, cell wall thickness, mean cell area, and number of missing cell walls.

Crumb color can be determined by chrommameter method (L*∗*,a*∗*,b*∗* values) [16, 38, 70] or computerized image analyses such as Photoshop system, which may facilitate not only a methodology for measurements of uneven coloration, but also it can be applied for the assessment of many other attributes of whole appearance as well [16]. Tlapale-Valdivia et al. [38] have mentioned that the crumb color can be mainly affected by both kneading and fermentation procedures and also by the fineness and homogeneity of the crumb grain [13].

## 6. Crumb Staling

Staling has been defined as a term that indicates decreasing consumer acceptance of bakery products caused by changes in crumb properties, except those resulting from the action of spoilage organisms [29, 82]. The most widely used indicator for staling can be considered as the increment of crumb firmness which is the parameter that is most commonly identified by the customers [9, 16, 70, 83].

Usually, leavened baked products begin to undergo deteriorative changes commencing with removal from the oven [82, 84]. The mechanism of crumb staling is more complex, more important, and less understood [1, 29]. Most of the sources identify the retrogradation (increasing crystallinity caused by cross linkages of starch molecules) of amylopectin as the main reason for staling [1, 37, 48, 82, 85–88]. Apart from the increment of starch crystallinity and opacity, increase in crumb firmness, changes in flavor, decrease in water absorption capacity, amount of starch and enzyme susceptibility of starch, and changes in X-ray diffraction pattern scan can also occur due to staling [1, 29, 48].

Moisture migrations can be also involved in staling process [11, 29, 82, 87]. This has been described in literature as the reversal in the location of water; therein water migrated to starch from gluten during baking and may return back to gluten proteins during storage [29, 82]. And also water may migrate from the crumb to the crust when the evaporation from the crust is prevented. This may cause the crust to gradually increase the leatheriness and remain soft while reducing the total moisture content in the crumb [29, 82, 89]. Usually, moisture in bread crumb acts as a plasticizer, where the reduction of crumb moisture content due to crumb staling can lead to formation of hydrogen bonds among the starch polymers or between starch and the proteins yielding increased crumb firmness [89, 90].

The staling rate can be affected by product size, moisture content, production process (e.g., baking time and temperature [1, 90]), packaging [89], and the storage temperature [29, 82]. As an example, lower storage temperatures (such as –1, 10, and 21°C) can accelerate starch recrystallization of the crumb compared with the storage at a warm room temperature (such as 32 and 43°C) [29, 82]. According to Slade and Levine [91] 4°C (refrigerator temperature) is the single optimum temperature that balances nucleation and crystallization and that the melting temperature involved implicates amylopectin as the polymer crystallizing. The effect of baking temperature on bread staling has been stated by Giovanelli, Peri, and Borri [90]. According to that, bread baked at lower temperatures (e.g., under slight vacuum to achieve crumb cooking at temperatures < 100°C) stales at a slower rate in terms of starch retrogradation [90].

Staling rate or crumb firming can be influenced by crumb structure, which is closely related to the gluten content, degree of starch gelatinization, and moisture redistribution [88]. This crumb firming kinetics can be applied to modified Avrami model (7) [85, 87, 92] based on starch retrogradation [87] where *θ* represents the recrystallization still to occur and *T*_*∞*_, *T*_0_, and *T*_t_ represent the bread hardness at *∞* time, bread hardness at zero time, and bread hardness at t time, respectively. “k” is a rate constant (constant time to compare bread hardening rate) that represents the parameters characterizing the crystallization process, and “n” is the Avrami exponent that is related to the crystal shape, way of crystallites nucleation, their subsequent growth, and the time dependence of the nucleation process [85, 87, 92]. Crumb firming kinetics may strongly depend on both “k” and “n”. As an example, crumbs with low “n” and/or “k” and/or “*T*_*∞*_” values may indicate slow crumb firming kinetics [92].

(7) $$\begin{array}{c} \theta =\frac{T_{\infty }-T_{t}}{T_{\infty }-T_{0}}=e^{{-kt}^{n}} \end{array}$$

Additionally, the degree of staleness can be analyzed by different methods [29]:

- Compression stress-strain curves and then determining Young's module
- Thermal analysis (including differential scanning calorimetry (DSC) and differential thermal analysis (DTA), thermogravimetric analysis (TGA), thermomechanical analysis (TMA), and dynamic mechanical analysis (DMA)) that provide basic information on starch retrogradation. As an example, DSC can measure the enthalpy associated with amylopectin recrystallization and monitors the progressive magnitude of staling endotherm [83]
- Infrared spectroscopy (Fourier transform infrared (FTIR) spectroscopy and near-infrared (NIR) spectroscopy)
- Magnetic resonance imaging (MRI)
- X-ray crystallography to measure the crystalline nature of the starch in the system
- Microscopy (transmitted light and polarized light microscopy can be used to monitor changes in starch granules from crumb before and after staling and confocal laser scanning microscopy to investigate the changes in starch granules in crumb during staling and electron microscope)
- Sensory evaluation

Several studies have shown that the total increment in crumb firmness may depend on bread specific volume, where higher crumb firmness may result in a product with lower specific volume [87]. According to study done by Błaszczak et al. [88], some properties like hardness and gumminess have increased while elasticity, cohesiveness, and volume recovery coefficient had decreased during the time of storage of bread samples. Additionally Błaszczak et al. [88] have showed that the crumb pores may become smaller and round during staling. Nussinovitch et al. [50] had proved that the crumb loses its elastic properties over time due to crumb staling. According to their research, the greatest loss occurs during the first 24 hours after baking and the loss had been drastic beyond that point. At a lower deformation, the damage can be slight or moderate and thus some recoverable work can be observed and its relative change during storage had been observed.

Literature states several studies that have been focused on the application of different additives and enzymes for extending the shelf-life of the leavened baked products by retarding the staling process [83]. Incorporation of surfactants such as monoglyceride, sodium stearoyl lactylate, and sucrose esters has been proved as a source that retards staling effect by retarding amylopectin retrogradation and by blocking moisture migration from gluten to starch [29, 86, 93]. Additionally, enzymes like *α*-amylases, hydrolases of non-starch polysaccharides [29, 83, 85, 88], proteases and lipases [29], emulsifiers like sodium/calcium stearoyl lactylate, mono/diglyceride [83], and hydrocolloids/gums [29, 68, 83] also have been applied to retard staling process (more details are described under “methods to improve porous crumb structure development and retard crumb staling”).

Freezing and frozen storage conditions can also have great influence on the quality and shelf-life of leavened baked products [1, 83, 94, 95]. The production of frozen dough requires flour with higher gluten quality than that used in conventional bread making processes as well as freeze-tolerant yeasts [83, 95], because, during freezing and frozen storage, the number of viable yeast cells decreases [83, 95, 96] and a reducing compound named glutathione is released as a consequence. This compound can break down the disulphide bonds among proteins leading to a weakening effect on the gluten. The weakening of the gluten network leads to increase in the proofing time as well as reduction in the oven spring and the dough resistance to stress conditions. This can result in the crumb to be lower in volume [48, 83, 95, 96] and coarse in texture with large and nonuniform air cells [95]. Additionally, this process is also reported to increase the fermentation time as well [48, 95, 96]. Rosell and Gómez [95] have described the applications of certain additives for improving the quality of bread prepared from frozen dough. According to that, application of additives such as gluten, emulsifiers, and hydrocolloids can improve the product volume and can improve the stability during frozen storage due to the water retention capacity of hydrocolloids.

Certain resent studies have moved from frozen dough to partially baked bread, called part-baked bread or prebaked bread [83, 95]. Many researches have mentioned that freezing prebaked bread is a better method to improve the shelf-life of the bread while keeping the bread quality similar to fresh products [48, 83, 94, 95]. According to Novotni et al. [89], partially baked bread has longer oxidative stability than fully baked frozen bread. One of the major problems of the part-baked and frozen bakery product is crust flaking. This relates to mechanical damage that occurs due to the intense thermomechanical shock during chilling–freezing and final baking [48]. According to the research conducted by Bácenas and Rosell [94], moisture content of the prebaked breads has been reduced while increasing the hardness with the increment of frozen storage. In addition, long times of frozen storage had been shown to be associated with greater aging rates. The physical damage of the prebaked bread during freezing has been caused by the progressive growing of the ice crystals which has been reported to be the main responsible cause for the quality loss and the greater speed rate of aging.

## 7. Methods to Improve the Porous Crumb Structure Development and Retard Crumb Staling

According to Seibel [97] and Jongh [98], pregelatinized flour and/or emulsifiers when working with composite flour with or without wheat flour, monoglycerides (0.5 - 1.0%), calcium and sodium stearoyl lactate (CSL and SSL) at a dose of 0.5-1.0% (flour basis), and binding agents (CMC, guar gum, carob gum, pregelatinized potato starch) can be applied to obtain better leavening and porous crumb structure and antistaling properties.

Food hydrocolloids or gums can be included in leavened baked products for diverse purpose and act as binding agents, such as gluten substitutes, to improve texture, to slow down the starch retrogradation, to increase moisture retention, and to extend the overall quality of the product throughout the storage period by decreasing the moisture loss consequently retarding the crumb hardening [23, 57, 68, 69]. As described by Collar et al. [57], the aforementioned qualities can be obtained even by using small quantities (<1% (w/w) in flour) of hydrocolloids.

Certain examples can be given for the application of several hydrocolloids in leavened baked products. Among them, hydroxypropylmethylcellulose (HPMC) and xanthan gum are the most widely used hydrocolloids to obtain better crumb properties (especially in gluten-free products) [80]. Additionally, guar gum, carboxymethylcellulose (CMC), locust bean gum, and agarose can also be used to improve the porous crumb structure of gluten-free bread formulas [99]. Anton and Artfield [23] and Bhol and Bosco [51] have cited Gan et al. [100] for their research findings regarding the application of HPMC, CMC, and guar gum in 50:50 wheat flour: rice flour formulation. According to that, HPMC at 1.7% and CMC at 0.4% have produced better bread characteristics than the application of guar gum at 0.7%. HPMC can preferentially bind to starch granules [68] and result in a rigid yet well porous and soft crumb texture with higher volume, improved sensory characteristics, and an extended shelf-life by providing the necessary viscosity of the dough to trap leavened gas during fermentation [23] and also retarding starch retrogradation by inhibiting the migration of water through interaction with starch molecules [29, 68]. Additionally, Eduardo, Svanberg, and Ahrné [68] have cited the findings of Shittu, Aminu, and Abulude [101] as the application of xanthan gum (1% from the composite flour weight) can improve dough handling properties, loaf specific volume, and crumb softness when incorporated into breads with composite cassava-wheat formulations.

Emulsifiers, pentosans, enzymes, or combinations of these can also be used as binding agents to improve the porous crumb structure and to obtain antistaling effects [69]. Emulsifiers are widely used in commercial bread formulas [68] to strengthen the dough that mainly interacts with gluten proteins, to improve gas retention capacity, and to soften the crumb as well as to retard staling effect [1, 68, 95]. The research carried out by Onyango, Unbehend, and Lindhauer [69] resulted in the fact that crumb hardness as well as staling rate can be decreased with the increase of the emulsifier concentration (better results have been observed for application of emulsifiers in 2.4% w/w flour wet basis than 0.4% w/w flour wet basis). Several examples for the emulsifiers incorporated in bread formulation include monoglycerides, sodium stearoyl lactylate (SSL), and diacetyl tartaric ester methyl (DATEM) [95].

The most frequently used enzymes to obtain better crumb structure are the *α*-amylases from different origins (cereal, fungal, and bacterial) to increase product volume and to improve crumb grain properties, crust, and crumb color and also have a contribution to flavor development [29, 85, 88]. Błaszczak et al. [88] have found that the addition of fungal (0.0014 g/100 g of flour) and bacterial *α*-amylases (0.03 g/100 g of flour) can have a substantial effect on starch behavior during dough fermentation, bread baking, and staling. In particular, addition of these enzymes had shown improved structural changes in the starch–protein matrix showing an antistaling mechanism [57, 88]. Hydrolases of non-starch polysaccharides (such as cellulose, xylanase, *β*-glucanase) are considered as another group of enzymes that can also be applied to improve properties of porous crumb structure [29, 85]. According to a research carried out by Haros, Rosell and Benedito, [85], addition of hydrolases of non-starch polysaccharides (the remaining enzyme activity in each flour as follows: cellulase treated flour (CEL) 85.5 mU/g of flour, xylanase treated flour (XYL) 2.9 mU/g of flour, and *β*-glucanase treated flour (GLUC) 1.2 mU/g of flour) had resulted in a product with lower crumb hardness, gumminess, and chewiness and reduced staling effect by reducing the initial crumb firmness and the kinetics of the firming process during storage. Skendi et al. [70] had found that bread prepared with barley *β*-glucan isolates (1.00 x10^5^, BG-100; lower molecular weight and 2.03 x10^5^, BG-200; higher molecular weight) resulted in a product with increased specific volume and reduced crumb firmness (especially the BG-200 sample).

Naturally extracted food gelling sources can also be used to improve the porous crumb structure of leavened products. According to the study done by Navaratne [79], bread samples containing* Davulkurudu* leaf (DKL) extract (the extraction of 10:100 leaves:water ratio had been incorporated until the dough moisture content reached to 58%) had given a better and well-developed porous crumb structure with reduced bulk density and better sensory attributes. Apart from that, staling process has also been reported to be retarded by 6 to 8 hours.

According to Różyło et al. [102], the enrichment with algal protein can also improve the rheological properties of dough, increase the gas retention capacity, and thereby lead to increase in product volume (4% per flour basis). Apart from that, the addition of algae had shown a positive influence on increasing elasticity and reducing the hardness of the crumb and reducing the degree of staling of gluten-free leavened products, which could have been found as a result of the presence of natural hydrocolloids in algae.

Garimella Purna et al. [37] had incorporated waxy wheat flour (15%, 30%, and 45% flour basis) into bread formulation and obtained a product with more open and porous structure (due to excessive swelling of waxy starch), high product volume, and softer texture (due to the combination of less amylose and more soluble starch from amylopectin). But the method had not retarded the staling process (due to rapid retrogradation of amylose in the initial stages of cooling and slow retrogradation of amylopectin for further firming the crumb). Additionally, they have mentioned that a significant post-bake shrinkage can be occurred in formulations with higher levels (>30%) of waxy wheat flour.

Researchers have found that the dough kneading and proofing have a vital effect on the quality of the crumb structure [44]. Poorly kneaded product had resulted in lower crumb brightness, fewer cells/cm^2^, thicker cell walls, and larger gas cells when compared with optimally mixed dough. Overkneading also resulted in lower quality product. Zghal, Scanlon, and Sapirstein [44] found that overproofing may lead to causing reduced cell wall thickness, increased cell size, and higher void fraction causing the crumb to be harder. And also it may lead to an increase in cell coalescence that results in losing gas cell walls.

## 8. Conclusion

Dough ingredients and processing conditions have a vital effect on the development of porous crumb structure of leavened foods. Certain modifications of those factors such as the addition of certain additives like hydrocolloids/gums, enzymes, and emulsifiers can impact the properties of porous crumb structure and crumb staling. Porous crumb structure is a heterogeneous complex structure and hence the crumb microstructure is strongly affecting the mechanical and sensorial properties of the final product. Well-developed porous crumb structure has the ability of retaining more leavened gas resulting in a product with increased volume and reduced crumb hardness.

## Figures and Tables

**Table 1:** List of examples of image processing software.

Image processing software	Detail	References
ImageJ	version 1.29, Natl. Inst. of Health, Bethseda, Md., U.S.A	Lassoued et al., [3]
		Bajd and Serša, [21]
		Tlapale-Valdivia et al., [38]
	http://rsb.info.nih.gov/ij or https://imagej.net/	Pérez-Nieto et al., [17]
		Curic et al., [48]
		Scheuer et al., [67]

SigmaScan®Pro	Version:5.50.4522.1800IC by Drug discovery Online	Romano et al., [12] Tlapale-Valdiviaet al., [38]
	http://www.sigmaplot.co.uk/products/sigmascan/sigmascan.php	Angioloni and Collar [16]

MATLAB	The MathWorks Inc.,	Gonzales-Barron and Butler [13]
		Bajd and Serša, [21]
	Natick, MA, USA	Shehzad et al.,[22]
		Rouillé et al.,[30]
	https://in.mathworks.com/	Verdú et al., [18]
		Eduardo, Svanberg and Ahrné [68]

Gebäckanalyse	Ver 1.3c 1997/98 program (Hochschule Ostwestfalen Lippe, Germany	Onyango, Unbehend and Lindhauer [69]

Labview	Vision Assistant 2009, National Instruments, USA	Che Pa et al., [5]

UTHSCSA	Version 2.0, University of Texas Health Science Centre, San Antonio, Texas	Skendi et al., [70]
ImageTool programme		
